# *In vivo* quantification of mechanical properties of caudal fins in adult zebrafish

**DOI:** 10.1242/jeb.171777

**Published:** 2018-02-15

**Authors:** Sahil Puri, Tinri Aegerter-Wilmsen, Anna Jaźwińska, Christof M. Aegerter

**Affiliations:** 1 Physik-Institut, University of Zurich, Winterthurerstrasse 190, CH-8057 Zurich, Switzerland; 2 Institute of Molecular Life Sciences, University of Zurich, Winterthurerstrasse 190, CH-8057 Zurich, Switzerland; 3 Department of Biology, University of Fribourg, Chemin du Musée 10, CH-1700 Fribourg, Switzerland

**Keywords:** *Daniorerio*, Flexibility, Stiffness, Experimental biophysics, Hydrodynamics, Locomotion

## Abstract

The caudal fins of adult zebrafish are supported by multiple bony rays that are laterally interconnected by soft interray tissue. Little is known about the fin's mechanical properties that influence bending in response to hydrodynamic forces during swimming. Here, we developed an experimental setup to measure the elastic properties of caudal fins *in vivo* by applying micro-Newton forces to obtain bending stiffness and a tensional modulus. We detected overall bending moments of 1.5×10^−9^–4×10^−9^ N m^2^ along the proximal–distal axis of the appendage showing a non-monotonous pattern that was not due to the geometry of the fin itself. Surgical disruption of the interray tissues along the proximal–distal axis revealed no significant changes to the overall bending stiffness, which we confirmed by determining a tensional modulus of the interray tissue. Thus, the biophysical values suggest that the flexibility of the fin during its hydrodynamic performance predominantly relies on the mechanical properties of the rays.

## Abstract (precis)

**Summary:** The quantitative *in vivo* determination of the zebrafish caudal fin's main constituents (bony rays and interray tissue) shows that flexibility is dominated by the elastic properties of the bony rays, whereas the elastic properties of the interray tissue co-define the fin's complex 3D deformation during swimming and will also be needed as a crucial input for hydrodynamic simulations.

## INTRODUCTION

Motion and the corresponding propulsive forces of flapping fins interacting with water flows have been a long-standing interest in biomechanics research (Iosilevskii, 2016; Borazjani et al., 2013; Witt et al., 2015; Esposito et al., 2012; Novarti et al., 2017; Dabiri et al., 2014; Nguyen et al., 2017; Fang-Bao et al., 2014; Noca et al., 1997; Verma et al., 2017). The shape, position and flexibility of fins display a high variability among fish taxa that accounts for a broad diversity of the locomotor functions. Nevertheless, the principal morphological organization of the fin dermoskeleton is evolutionarily conserved among all ray-finned fishes. The main material of the fin consists of an array of similar bony elements (lepidotrichia) interconnected by soft tissue. Little is known about the biophysical properties of the (caudal) fin's anatomical structures in relation to hydrodynamic forces. Knowledge of the mechanical features of the functional tissue is necessary to understand how a deforming fin interacts with the surrounding fluid to generate swimming forces. Furthermore, characterization of the physical parameters of the tissues can be relevant for interdisciplinary studies on the interplay between hydrodynamic forces and genetic pathways regulating fin patterning and growth. Such studies require knowledge of the extent of forces acting on the fins over time and space. This in turn is highly facilitated by carrying out numerical simulations of swimming fish and their flapping fins. For this purpose, information about the elastic properties of fins is necessary to perform accurate simulations coupling elasticity and hydrodynamics that allow extraction of realistic forces working on the fin.

From a genetic perspective, zebrafish are the best-studied fish. The caudal fin of adult zebrafish is the main appendage used for propulsion while swimming. Its shape and size are closely associated with the overall swimming performance of the fish. Anatomically, this fin can be defined as a non-muscularized appendage that is stabilized by 16–18 rays that are further subdivided into segments and occasionally bifurcate. The rays are spanned by soft interray tissue (Akimenko et al., 2003; Pfefferli and Jazwinska, 2015) (Fig. S1). Fin growth is achieved through the sequential addition of new ray segments at the tip, which, once formed, can become progressively thicker (Goldsmith et al., 2006; Brittijn et al., 2009; Marí-Beffa and Murciano, 2010).

While swimming behaviour has been studied in zebrafish at different developmental stages (Müller and Van Leeuwen, 2004; Danos and Lauder, 2007; McHenry and Lauder, 2005), the mechanical properties of the fins, including the caudal fin, are not known. In other fish, the stiffness and bending properties of dissected single rays *ex situ* have been measured (Videler, 1977; Alben et al., 2007; Flammang et al., 2013). However, the methods applied cannot be used directly, as zebrafish are much smaller and thus the bending stiffness is expected to be lower by several orders of magnitude. This poses additional experimental challenges on the sensitivity of the force sensor, the accuracy of the applied deflection and the prevention of spurious movements. In addition, we aimed to measure properties in living fish, thus requiring anaesthetic procedures and a holding device.

Here, we describe the apparatus we developed to measure *in vivo* bending stiffness profiles along the proximal–distal axis of caudal fins. We show that bending stiffness has a peak along this axis, which cannot be readily explained from geometry itself. In addition, we surgically removed interray tissue and found that this did not observably affect bending stiffness. Finally, we determined an estimate of the tensional modulus of the interray tissue from bending only parts of the fin, whereby the tissue is stretched.

## MATERIALS AND METHODS

### Animal procedures

Wild-type AB (Oregon) zebrafish, *Danio rerio* (F. Hamilton 1822), aged 6–12 months were used in this study. The fish were maintained at 26–27°C (Westerfield, 2007). The exact sample size for each experiment is given in the corresponding figure legends, and was chosen to ensure the reproducibility of the results. Fish were anaesthetized with 0.5 mmol l^−1^ tricaine methanesulfonate (MS-222 ethyl-*m*-aminobenzoate, Sigma-Aldrich, St Louis, MO, USA) in system water that was freshly prepared from 15 mmol l^−1^ buffered stock solution, which was stored in the dark at 4°C.

**Fig. 1.**
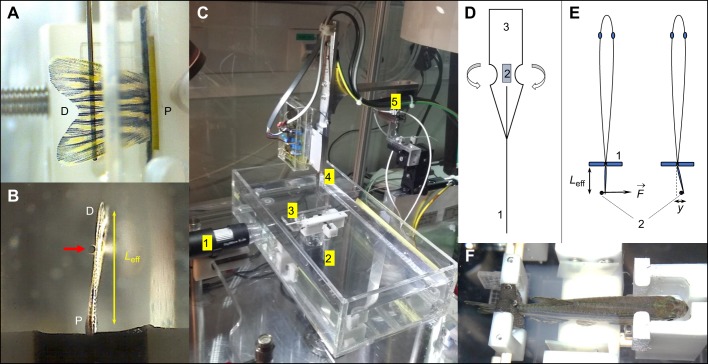
**Overview of the setup showing the basic principle of measurement.** (A) Side view of a lined force attack from the deflection pin onto the fin surface (D, distal end of the fin; P, proximal end of the fin). (B) Bottom view showing the pin (red arrow) at a certain effective beam length from the fixation (black foam pads). (C) Overall view of the measurement setup showing the water basin, two camera positions (1 and 2) as in A and B, respectively, a fish-holding device (3), as also shown in F, and the sensor construct (4) mounted onto piezo-based positioners (5). (D) Design of the sensor construct with strain gauges (2) mounted onto a spring sheet (3), with the deflection pin (1) that leads to the base of the strain gauges to increase the effective bending moment together with the specialized geometry of the spring sheet (3). (E) Measurement principle illustrating the deflection pin (2) applying a force (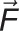) at a particular proximal–distal position (effective beam length, *L*_eff_) leading to a particular displacement *y*. The fish peduncle is held by two opposing foam pads (1), as also shown in B. (F) Zebrafish-holding device showing an anaesthetized adult specimen.

During fin bending measurements, each fish was placed in a custom-built holding device (Fig. 1; Fig. S2) and immersed in the measurement basin containing 0.4 mmol l^−1^ MS-222. The animals were continuously monitored for regular opercular motions (Fish et al., 2008) to avoid mortality risks. Each measurement cycle of one caudal fin including preparatory tasks takes between 1 and 1.5 h. After completing one measurement cycle, the fish were immediately transferred to a recovery basin containing system water. Once the fish restarted normal swimming, they were transferred into the regular system. Animal procedures were approved by the cantonal veterinary office of Fribourg, Switzerland.

To measure caudal fins lacking a mechanical connection between each ray, we surgically disrupted the connective interray tissue with a 3 mm dissecting knife (Roboz, Gaithersburg, MD, USA), using an intubation-based anaesthetic delivery system (Xu et al., 2015) placed under a stereo microscope (Zeiss, Oberkochen, Germany). Before transferring the fish to the measurement chamber as described above, each fish was allowed to recover for at least 30 min to reduce overall stress levels.

### Histological staining of fin sections

Fins were fixed in 4% paraformaldehyde in PBS, dehydrated, and embedded in tissue freezing media (Tissue-Tek O.C.T.; Sakura, Alphen aan den Rijn, The Netherlands) as described previously (König et al., 2018). Cryosections were cut at a thickness of 12 µm, rehydrated and stained with Mayer's Haemalum for 12 min. The nuclear staining was differentiated for 5 s in 0.37% HCl prepared in 70% ethanol, and the slides were washed in tap water for 10 min. The staining of proteins was obtained by incubation for 10 min in 0.1% Eosin Y solution in water with a drop of acetic acid, followed by a rapid wash in water. The sections were dehydrated in a water/ethanol series, cleared in xylol, and mounted in Entelan medium (Merck, Kenilworth, NJ, USA).

### Setup design

An overview of the setup is shown in Fig. 1 and the corresponding computer-aided design (CAD) model is shown in Fig. S2. In order to obtain the necessary sensitivity for the measurement of the small bending stiffness of zebrafish fins, we followed a two-pronged approach. First, the deflection of the fin had to be measured with great accuracy, which is why we used a calibrated piezo-driven stage (SmarAct, GmbH, Oldenburg, Germany) displacing a 0.4 mm deflection pin (Fig. 1D) attached to the force sensor construct with sub-micrometre precision. Second, we measured the sensitivity of the force sensor, using two linear strain gauges (350 Ω; OMEGA Engineering, Manchester, UK) attached on either side of a custom-fabricated steel spring sheet with a thickness of 0.03 mm (Fig. 1D). We used a pair of strain gauges in order to prevent systematic drift due to, for example, temperature changes (strain gauge configuration: half-bridge type 2). The strain gauges were internally wired as a Wheatstone bridge. A signal amplifier (INA101, Burr Brown, Tucson, AZ, USA) was used to improve the electronic readout. The analog-to-digital conversion was managed by a USB-bus (16-bit, 400 kHz; National Instruments, Austin, TX, USA) to convert voltage output into a direct force readout using custom-made control software (LabVIEW, National Instruments). The thickness and geometry of the spring sheet near the strain gauges (Fig. 1D) was chosen in order to reach an optimized signal-to-noise ratio for the envisaged force range near 100 µN.

Finally, this force sensor had to be combined with the setup to minimize movements of the deflection tip. Therefore, we used small stepping distances to reduce inertial vibrations of the spring sheet resulting from piezo-based motor activities. The anaesthetized fish was positioned in a holding device using synthetic foam pads, leading to reproducible positioning along the proximal–distal axis (Fig. 1; Fig. S2).

### Calibration of setup

The strain sensors were calibrated using five different high-precision weights (Mettler-Toledo, Columbus, OH, USA) exerting forces ranging from 0.2 to 5 mN. These weights were mounted at the end of the deflection pin horizontally. The voltage output for each calibration could be converted into a force unit and used in the custom-made software.

### Benchmarking

Before measuring caudal fins *in vivo*, we conducted measurements on three thin, rectangular cantilevers with known elastic moduli to validate the performance of our calibrated setup in the region of smaller forces and deflections. We performed several force–deflection measurements at various positions along the cantilevers (Fig. S3) to determine the bending stiffness *EI*, which, using the known geometry of the cantilever, was directly related to Young's modulus *E*, given the area moment of inertia *I* (Eqn 1) (Gere and Timoshenko, 1984):
(1)
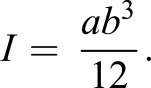
Here, *a* and *b* are the width and the thickness of the cantilever, respectively.

### Experiments and data collection

We measured the bending stiffness of caudal fins *in vivo* by applying a mechanical line force load (Fig. 1A) to deflect the fin by a specified distance (Fig. 1E). The resulting bending of the cantilever–pin construct containing the strain gauges was used to determine the applied force at a given deflection. In general, the dependence of the deflection of the cantilever, i.e. the fin, in response to such a force load *F* is described by the Euler–Bernoulli equation (Eqn 2):
(2)
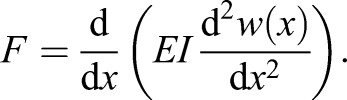
Here, the deflection is denoted by *w*(*x*), which changes along the proximal–distal axis, *x*, and *EI* is the bending stiffness, describing the resistance of the cantilever to bending. This bending stiffness contains a dependence on the material's property (Young's modulus *E*), as well as the geometry via the area moment of inertia *I*, given for a rectangular beam in Eqn 1. Solving the Euler–Bernoulli equation for the case of a constant *EI*, and setting the boundary conditions of being clamped at the origin, i.e. *w*(0)=0 and *w*′(0)=0, and being able to freely deflect at the load position (*L*) by a distance *y*, i.e. *w*(*L*)=*y* and *w*″(*L*)=0, one obtains a dependence of the deflection on the force given by (Gere and Timoshenko, 1984; Vogel, 2014):
(3)
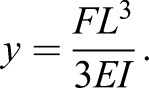
Given the slope of such a force–deflection curve, we could then calculate the bending stiffness (*EI*). In the fin, the geometric proportions are not constant along the proximal–distal axis, which implies that *EI* is a function of *x*, thus changing the solution of the Euler–Bernoulli equation above. However, for a linear change of the width and thickness of the cantilever, this can be approximately taken into account by taking an average of the length dependence of the area moment of inertia, using:
(4)
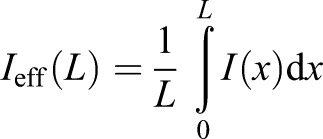
as a constant in the derivation of Eqn 3, where *I*_eff_ is the effective area moment of inertia.

In the determination of the force–deflection curves, we applied a 10 µN threshold force as a standard offset to ensure that the pin touched the fin surface equally (Fig. 1A) before starting a measurement. Furthermore, we did not exceed a deflection of 2 mm to avoid positional drift of the pin on the fin surface. This is because the pin can slip when the fin surface is curved too much during deflection measurements. We performed bending measurements along the proximal–distal axis at six distinct positions (see Fig. S4), returning to an unloaded situation after each positional measurement.

Measurements of the effective beam length, i.e. the distance from the fixation point to the loading point (Fig. 1B,E) were done using a digital USB microscope (dnt GmbH, Dietzenbach, Germany) and analysed in ImageJ (version 1.51h).

## RESULTS AND DISCUSSION

### Cantilever benchmarking

To test the calibration of the force sensors for small forces and to determine the bending stiffness of thin, soft cantilevers, we used two different cantilever materials for benchmarking. This approach yielded reproducible results (Fig. S3). The two materials used were steel, with a Young's modulus of 206 GPa, and polyimide, with a Young's modulus of 2–3 GPa. These cantilevers were highly linear in their bending properties and our force sensor could very reproducibly determine force–extension curves with a slope much below 1 N m^−1^ at forces near 100 µN. For the steel cantilever, we obtained an average modulus (±standard error, a measure of the experimental uncertainty) of 200±10 GPa (Fig. S3A). For polyimide, similarly, the data gave an average modulus of 2.0±0.3 GPa (Fig. S3B), consistent with previously published values (Haynes, 2016). This agreement was also found when measuring in water, whereby a value of 2.2±0.2 GPa was obtained (Fig. S3C). Thus, we did not observe a difference between the data obtained in air and water, consistent with the static nature of the measurements.

From these benchmarks, we concluded that our setup can measure bending stiffness in water at forces of 100 µN to an accuracy of at least 10%. These findings validate the suitability of our system for measuring the bending stiffness of live zebrafish caudal fins.

### *In vivo* measurements

**Fig. 2.**
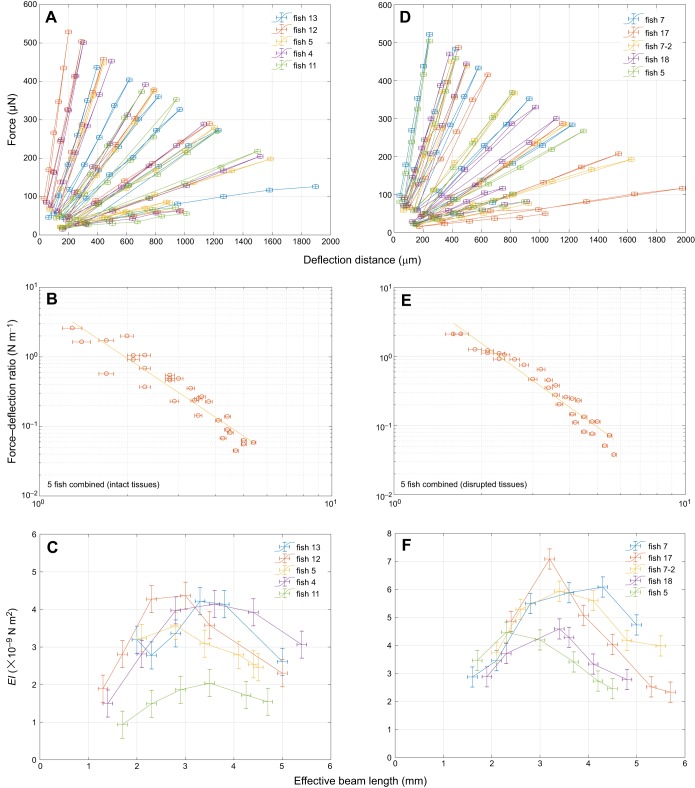
**Elasticity measurements for caudal fins.** (A-C) Data for intact fins; (D-F) data for disrupted fins. (A,D) Force–deflection curves for five fish at six positions, for fins with intact (A) and disrupted interray tissue (D), show a high degree of linearity for deflection distances up to 2 mm [error bars for deflection distance correspond to standard deviations (±20 µm), equally for force (±5 µN)]. (B,E) The ratio of force over deflection for all the curves in A and D as a function of the effective beam length on a double-logarithmic scale. The slope is consistent with the cantilever expectation (Eqn 3) of a cubic dependence. (C,F) Stiffness values (*EI*) along the proximal–distal axis of intact (C) and surgically disrupted fins (F). Error bars correspond to standard deviations (±0.1 mm) for the effective beam length, whereas error bars for stiffness (*EI*) were obtained from errors on force, deflection distance and beam length using error propagation.

**Fig. 3.**
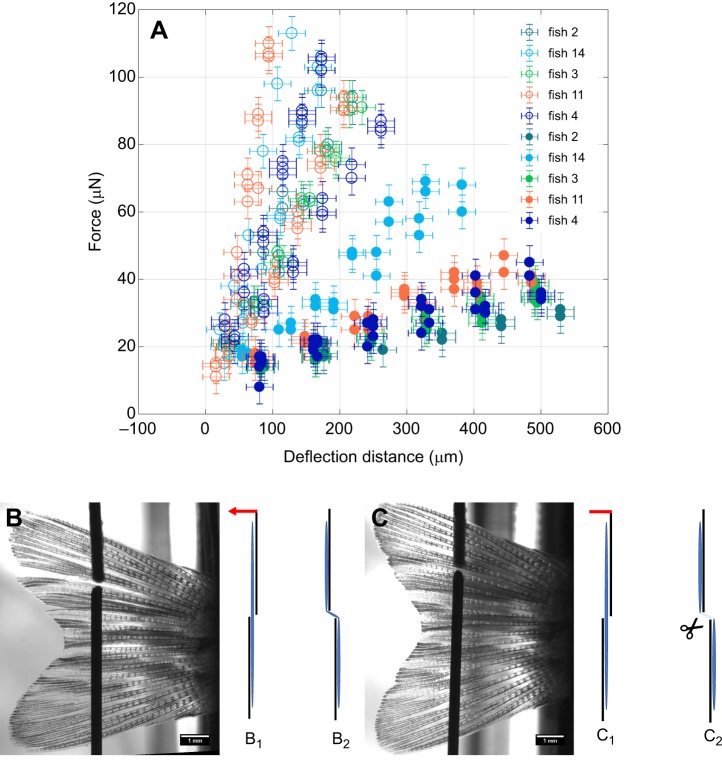
**Elasticity measurements for the interray tissues.** (A) Force–deflection curves for five fish. Open symbols indicate measurements performed on intact tissues (B), whereas filled symbols show the results for surgically disrupted tissues (C). Error bars for deflection distance correspond to standard deviations (±20 µm), equally for force (±5 µN). Measurements were performed at the same positions in each fin, once between the 4th and 5th ray (B,C) and once between the 6th and 7th ray (not shown). The illustrations B_1_, B_2_, C_1_ and C_2_ represent front views. B_1_ and C_1_ represent the starting position, before a deflection to the left (indicated by a red arrow) stretches the interray tissue (B_2_), resulting in the configurations B_2_ and C_2_, respectively.

After validation, we measured the bending properties of five adult zebrafish caudal fins at six distinct positions along the proximal–distal axis (Fig. 2A–C). The force–deflection curves revealed a high degree of linearity (Fig. 2A). This indicates that the fin acts as a linearly elastic medium in response to a bending load as required by Eqn 3. This is furthermore underlined as a characteristic cubic behaviour as depicted in Fig. 2B. Nevertheless, the dependence did not entirely reflect a uniform, elastic beam, as can be seen by determining the bending stiffness (Fig. 2C), where a peak was observed at ∼2–4 mm from the fixation. Such a position dependence is not unexpected given that the fin is not shaped as a rectangular beam and hence *I* (Eqn 1) changes with proximal–distal position, such that the bending stiffness *EI* will also change with position. This geometrical influence can be quantified in a simplified way using Eqn 4 based on morphological data from zebrafish fins (Fig. S1). The thickness of fins at the deflection positions decreases from 200 to 40 µm as determined with a digital calliper, which is also in agreement with the diameter of the rays obtained from live images (Fig. S4). With the increase in fin width at the same positions from ∼5 to 8 mm, Eqn 4 leads to a monotonically decreasing dependence of *I*_eff_ from 7×10^−16^ m^4^ to 3×10^−16^ m^4^, given a homogeneous shape similar to that of the zebrafish caudal fin (Fig. S5A). Considering that the bending stiffness is mostly determined by the rays, we determined the diameter of the rays and calculated their average *I*_eff_ values (Fig. S5B). Given the monotonic dependence of both these effective area moments of inertia on the length, we calculated the effective Young's modulus by dividing the effective bending stiffness by *I*_eff_, which consistently showed a peak (Fig. S5C).To disentangle the relative contributions to stiffness of the bony rays and the soft interray tissue, we surgically removed interray tissue (Fig. S6). When measuring the bending stiffness in these fins, we found that the influence of the interray tissue on the overall bending stiffness was negligible (Fig. 2D–F). Even though there was no influence of the interray tissue on the bending stiffness, its mechanical properties are still of great importance in determining the interaction between the fin and the water during propulsion. Therefore, we also used our setup to measure the tensional elasticity of the interray tissue. For this purpose, we modified the setup in order to bend not the entire fin but, rather, only one part (Fig. 3B,C). In addition to the bending force necessary for bending the upper part against the lower part, this approach also involves a force that stretches the interray tissue between two adjacent rays. These two forces can be disentangled by again disrupting the interray tissue between the two parts of the fin (Fig. 3C). The difference in elastic forces between the two force–deflection curves for intact fins and disrupted fins (Fig. 3A) corresponds to a force–extension curve of the interray tissue between the two adjacent rays that was severed in the second experiment (Fig. 3C). This stretching was uniaxial along the tissue in the direction of the deflection as a result of the geometry of the setup (Fig. 3B, B_2_). This yielded a linear force–extension curve with an average slope of *k*_intact_=0.4±0.1 N m^−1^ for intact tissue and *k*_cut_=0.08±0.04 N m^−1^ for disrupted tissue. In order to translate the difference between these two spring constants, Δ*k=k*_intact_−*k*_cut_, to an effective tensional modulus, we needed to take into account that the strain is given by ε=*y*/*d*, where *d* is the width of the stretched interray tissue and *y* is the deflection. Thus, the elastic stress is given by σ=*E*ε=*Ey*/*d* and hence the stretching force by *F=*σ*bw*=*Eybw*/*d*, where *b* is the breadth and *w* the thickness of the stretched tissue. Using this relationship from Hooke's law, we obtained a slope difference of the two averaged force–extension curves of Δ*k*=*Ebw*/*d* or, alternatively, a tissue modulus of *E*=*Δkd*/(*bw*). If we assume the width and breadth of the stretched tissue to be of similar magnitude, this simplifies to *E*=*Δk*/*w* and with a width between 50 and 100 µm from Fig. S1, we obtain an effective modulus of the interray tissue of *E*_tissue_=4±2 kPa. Note that the breadth has to be at least as large as the width, such that this estimate presents an upper bound for the modulus.

### Conclusions

Here, we have described an apparatus to measure the bending stiffness of fins of live zebrafish at the level of 10^−9^ N m^2^, as well as a method to determine the tensional elasticity of the soft interray tissue to the level of kPa. Previous measurements of bending stiffness of larger fish have reached a sensitivity of 10^−6^ N m^2^ (Flammang et al., 2013), which in that case was sufficient because of the larger dimensions of the fins and rays of bluegill sunfish. Given the diameter of bluegill rays of 1–2 mm compared with 0.1–0.2 mm for zebrafish, one would expect zebrafish rays to be more than four orders of magnitude more flexible than those of bluegill sunfish. As there are 16–18 rays making up the entire zebrafish fin, the bending stiffness of bluegill rays of 3.8×10^−6^ N m^2^ would indicate a bending stiffness for a zebrafish fin of between 2×10^−9^ and 5×10^−9^ N m^2^, in reasonable agreement with our measurements, which are for live fish.

However, within that range of stiffness values, we found that the zebrafish fin shows a non-monotonous dependence of bending stiffness along the proximal–distal axis. Comparing the stiffness data with morphological images of the individual fins (Fig. S1), it seems that the position of peak stiffness coincides with the position of the first bifurcation of the ray skeleton. This suggests that the formation of a bifurcation is associated with regulation of the overall mechanical properties of the fins.

However, when studying the locomotion of the fish, not only the fin's bending stiffness but also its tensile strength will be important. This may well be dominated by the material properties of the soft interray tissue, such that determination of the tensile strength of the interray tissue is also necessary for a complete description of the mechanical properties of the caudal fins determining the fish's locomotion. Using a deflection of only part of the fin, we have obtained an estimate of this tensile strength, of the order of a few kPa.

In order to elucidate interactions between genetics and hydrodynamics, it will be necessary to know more about the effects of single genes on the establishment of the fin's mechanical properties. Therefore, we envisage that our apparatus will be used to measure bending properties of different mutants. In addition, the extent of forces acting on the fins of swimming fish needs to be known in order to correlate gene expression patterns with hydrodynamic forces. For this purpose, coupled elasto-hydrodynamic simulations can be used, which need mechanical parameters such as those determined here as inputs. Therefore, we view these measurements as a first step in bridging the gap between genetics and hydrodynamics in the question of locomotion in zebrafish.

## Supplementary Material

Supplementary information
