# Supplementary material for: In vivo quantification of mechanical properties of caudal fins in adult zebrafish
Source: J Exp Biol. 2018 Feb 15;221(4):jeb171777. doi: 10.1242/jeb.171777 (PMC5868929; doi:10.1242/jeb.171777)
Supplement: Supplementary information [file jexbio-221-171777-s1.pdf]

Supplementary figures

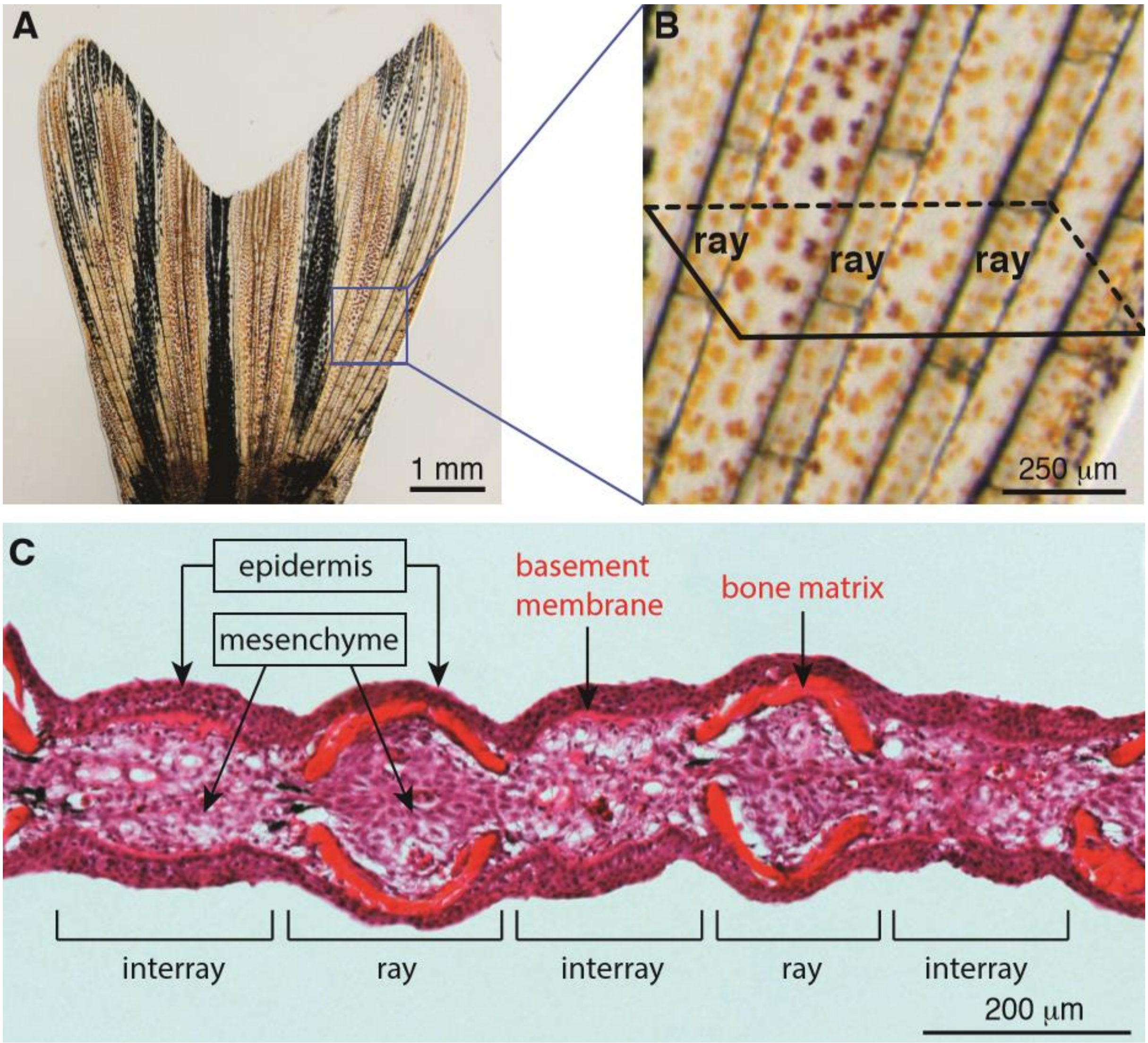

Fig. S1: The structural and histological organization of a caudal fin in adult zebrafish. (A-B) Live-imaging of the caudal fin. (A) The adult fin has a bi-lobed morphology, supported by an array of bones, which occasionally bifurcate. (B) Higher magnification of the framed area in (A) displays segmented bone-containing rays, called lepidotrichia, which are spanned by soft interray tissue. The schematic parallelogram depicts a transversal sectioning plane for histological analysis. (C) Transversal section of a fixed fin stained with Haematoxylin (nuclei, violet) and Eosin (cytoplasm, pink/magenta; extracellular matrix, red/orange). Rays are supported by a pair of concave bones underneath the epidermis. The interray tissue is devoid of skeletal elements and the epidermis is supported by the thickened basement membrane. The mesenchymal tissue fills the inner part of the fin, and it contains fibroblasts, extracellular matrix, nerves and blood vessels.

Journal of Experimental Biology • Supplementary information

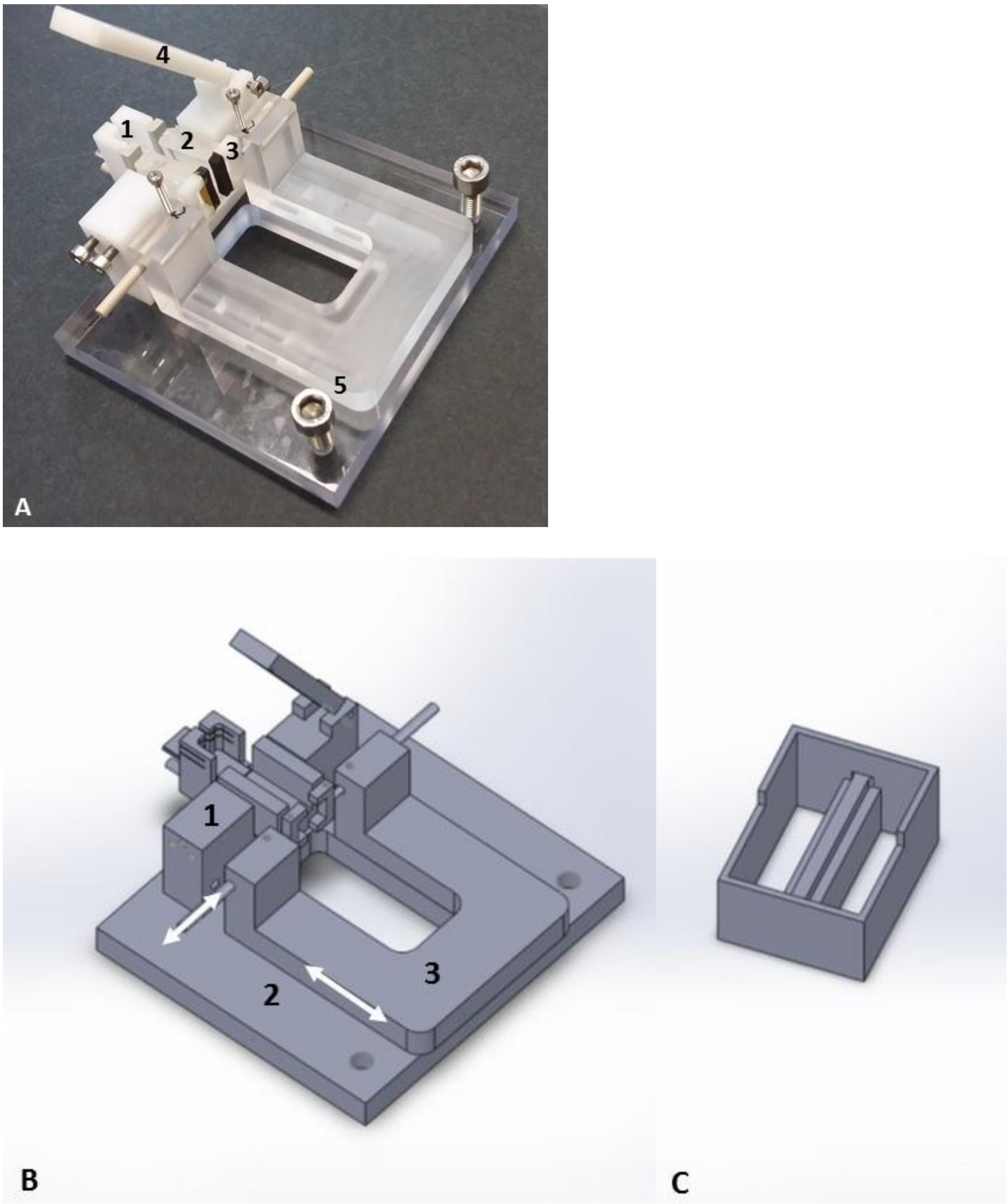

Fig. S2: Live image and CAD model of the fish holding device. (A) A head placement chamber (1) is shown as already indicated in Fig. 1, F, an agarose pad (2) to place the fish softly by adjusting the pad size by screws from the two opposing sides, two clips equipped by soft synthetic foam pads (3) to position the fin peduncle, a movable lid (4) to keep the agarose pad in place under water and finally two screws (5) serving as handling grips for transferring the device to the water containing basin. **(B)** A CAD (computer-aided design, software: SolidWorks Corp. 2016) model is shown with the 3 main components: fish holding module (1), base plate (2) with a sliding track (white arrow) to adjust for different fish sizes. A second white arrow indicates the movability of the peduncle fixation clips. For the entire holding device, non-toxic materials were used: (1) POM, (2) PMMA and (3) Macrolon (also see (A)). **(C)** A casting mold (material: POM) was used to fill in heated 1% (wt./vol.) agarose to reach a desirable shape and softness after cooling (A, (2)).

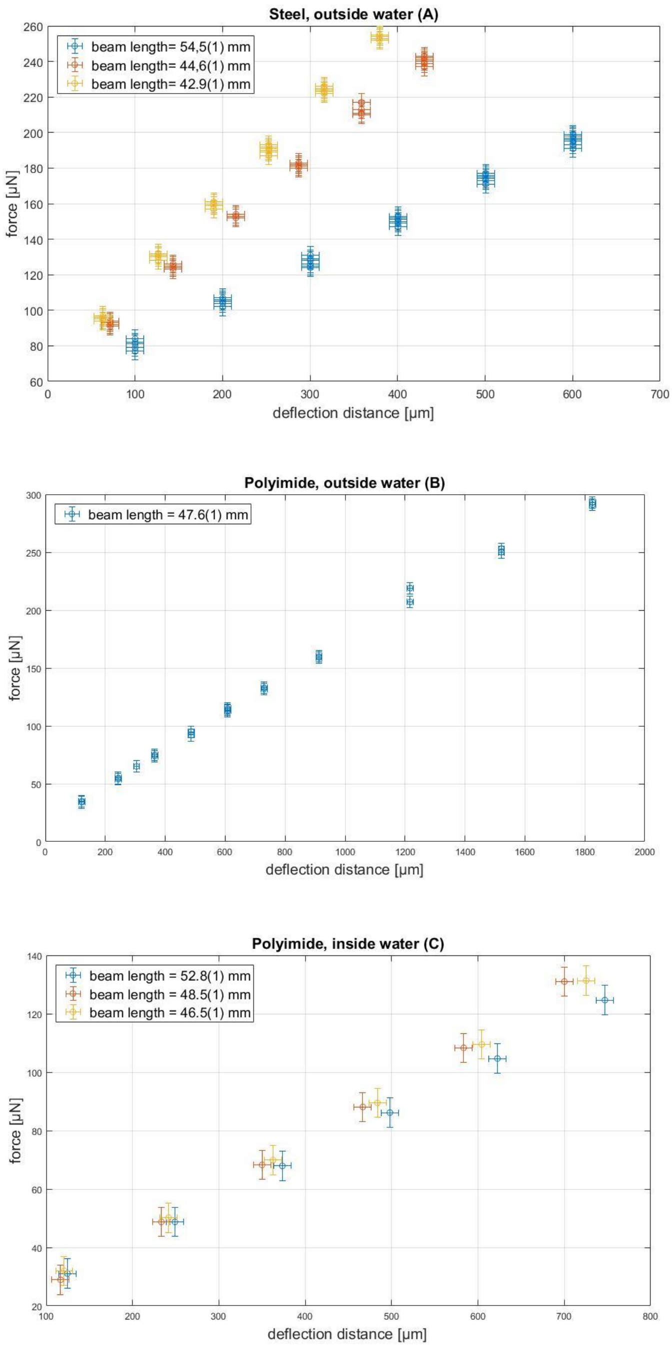

Fig. S3: Force-deflection curves for three cantilever benchmarking measurements. Steel outside water (A), polyimide outside water (B), polyimide inside water (C). The curves demonstrate a high degree of linearity for all deflection distances measured. The steel cantilever (A) was tested at 3 distinct positions as indicated, with increasing and decreasing loads. From the slope of the force-deflection curves and the deflection distance, the moduli determined are: 198(19) GPa, 194(15) GPa and 209(16) GPa. Cantilevers with smaller stiffness made of polyimide were also studied (B, C). This was measured both outside (B) and inside (C) of a water tank to test the situation comparable to live fish. The elastic moduli obtained are in this case: outside water (B): 2,0(3) GPa; inside water (C): 2,5(2) GPa, 2,4(1) GPa and 1,9(1) GPa for determinations at three distinct positions (error bars for deflection distance correspond to standard deviations (± 20µm), equally for force (± 5µN).

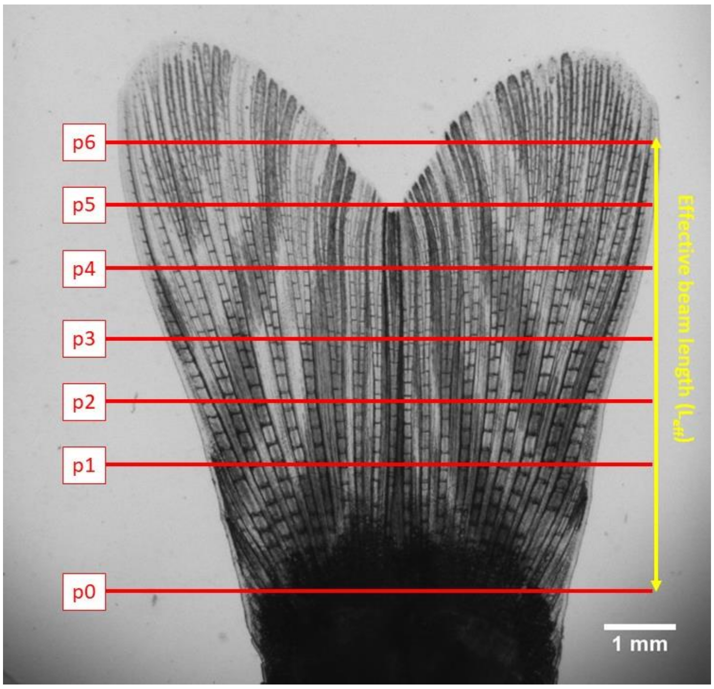

Fig. S4: Proximal-distal deflection positions in normal fins. The red lines (positions p1-p6) indicate the 6 deflection positions along the fin. A plane of ray bifurcations can be seen at about 3mm (p2-p3) from the fixation point (p0). The effective beam length (L_{eff}) is used in Eq.1.

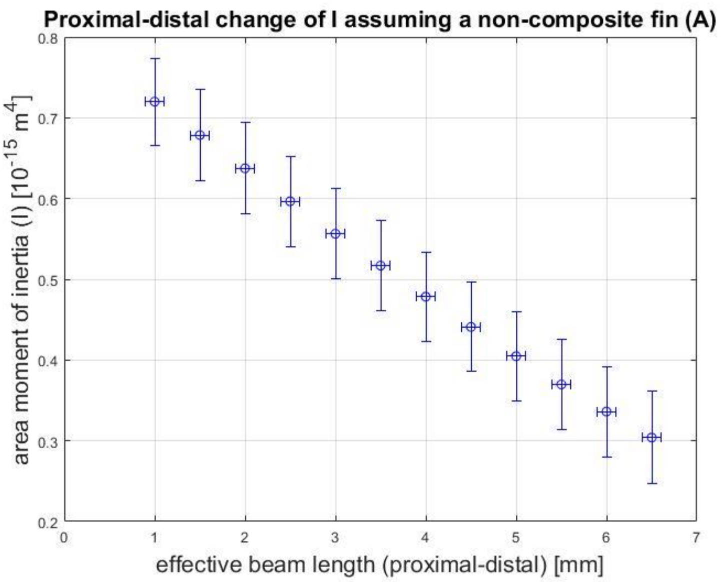

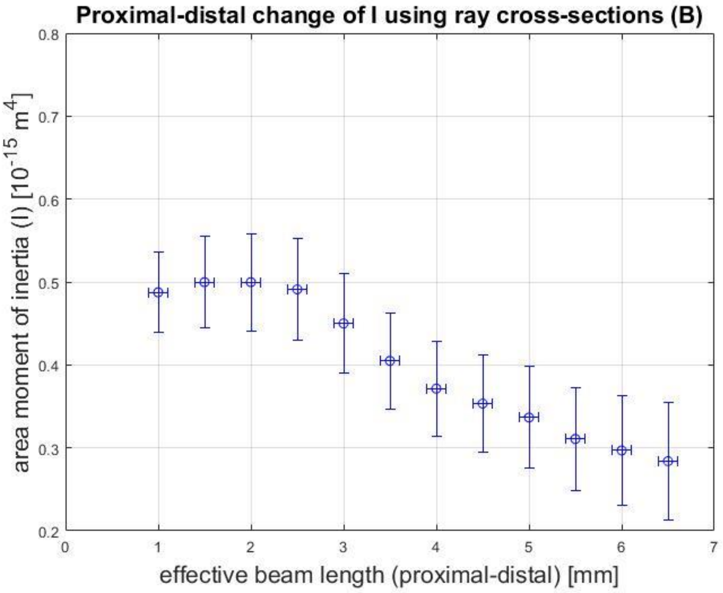

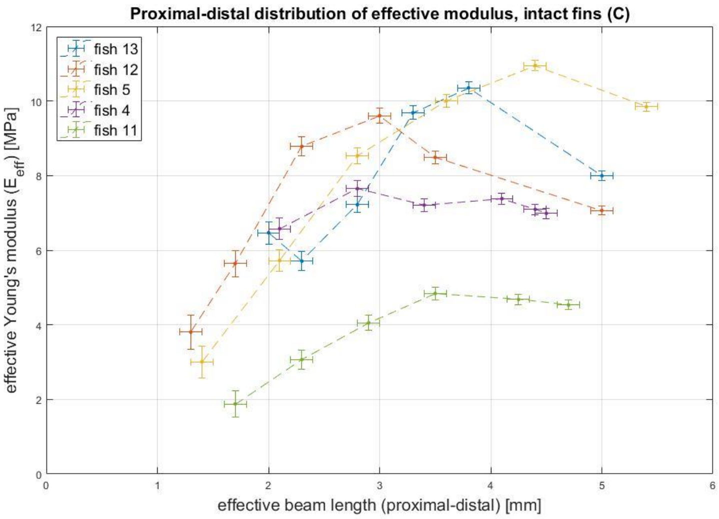

Journal of Experimental Biology • Supplementary information

Fig. S5: Proximal-distal distributions of two different area moment of inertia (*I*) are shown. Assuming a fin made of homogenous material and ignoring the contribution of single rays we show the geometric influence against the effective beam length (A) by calculating the influence of the changing width a(x) and thickness b(x) given by Eq. 4, which presents an approximate description. Thus, the variation of *I* reflects the shape of the fin. In (B) we show a similar calculation, taking into account only the rays and their change in thickness along the length of the fin for every ray and adding up all these contributions. The rapid slope change between 2-3 mm corresponds to the 1st bifurcation plane (Fig. S4). Error bars correspond to standard deviations (± 0.1mm) for the effective beam length, whereas error bars for both area moments of inertia (*I*) are obtained using error propagation. Using the effective area moment of inertia shown in (B), the bending stiffness presented in Fig. 2 C can be translated into an effective elastic modulus for the rays, which is shown here in (C).

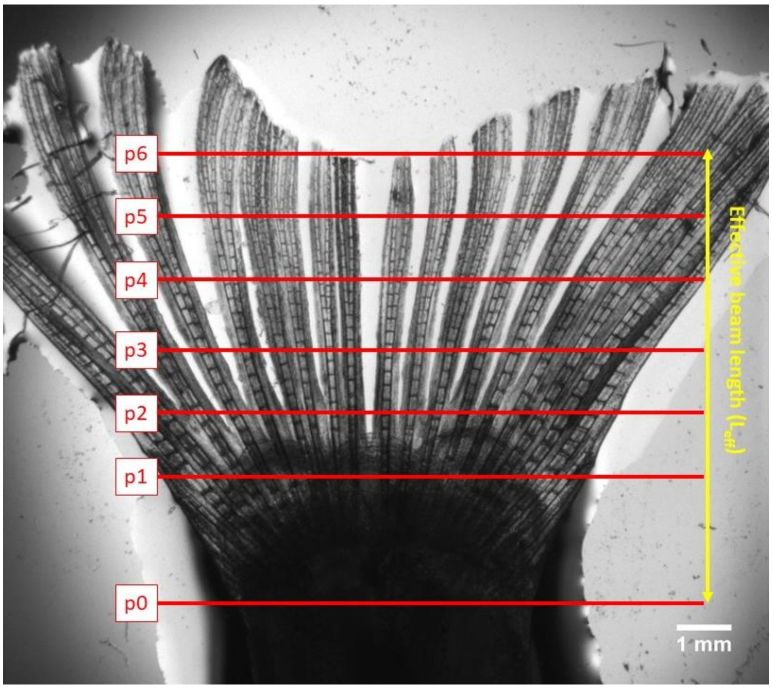

Fig. S6: Proximal-distal deflection positions in surgically disrupted fins. Shape of a zebrafish fin where the interray tissue has been surgically removed. The bending stiffness of such a fin is only determined by the stiffness of the rays.
